# The Peptide Hormone CNMa Influences Egg Production in the Mosquito *Aedes aegypti*

**DOI:** 10.3390/insects13030230

**Published:** 2022-02-25

**Authors:** Nia I. Keyes-Scott, Aryan Lajevardi, Kyle R. Swade, Mark R. Brown, Jean-Paul Paluzzi, Kevin J. Vogel

**Affiliations:** 1 Department of Entomology, The University of Georgia, Athens, GA 30602, USA; niaks@uga.edu (N.I.K.-S.); k.swade@comcast.net (K.R.S.); mrbrown@uga.edu (M.R.B.); 2 Department of Biology, York University, Toronto, ON M3J 1P3, Canada; aryanlajevardi@gmail.com (A.L.); paluzzi@yorku.ca (J.-P.P.)

**Keywords:** GPCR, insect endocrinology, reproduction

## Abstract

**Simple Summary:**

Hormones are important signaling molecules mediating insect reproduction that require specific receptors to transmit their signal across the plasma membrane of target cells. While the receptors many hormones bind to are known, some receptors are “orphans” with unknown ligands. The hormone CNMa was previously found to bind a specific receptor in fruit flies. Two copies of the receptor are found in mosquitoes, but only one gene for the hormone is present. We found that both receptors in the yellow fever mosquito are activated by CNMa. One of the receptors is expressed in mosquito ovaries, which to us implies that the hormone and its receptors may be involved in mosquito reproduction. We further found that injecting the peptide into females reduced the number of eggs that were laid. These experiments suggest that CNMa may be an important factor governing mosquito reproduction.

**Abstract:**

Mosquito reproduction is regulated by a suite of hormones, many acting through membrane-bound receptor proteins. The *Aedes aegypti* G protein-coupled receptors AAEL024199 (AeCNMaR-1a) and AAEL018316 (AeCNMaR-1b) were identified as orthologs of the *Drosophila melanogaster* CNMa receptor (DmCNMaR). The receptor was duplicated early in the evolution of insects, and subsequently in Culicidae, into what we refer to as CNMaR-1a and CNMaR-1b. AeCNMaR-1a is only detected in male mosquito antennae while AeCNMaR-1b is expressed at high levels in mosquito ovaries. Using a heterologous cell assay, we determined that AeCNMa activates AeCNMaR-1a with a ~10-fold lower concentration than it does AeCNMaR-1b, though both receptors displayed half maximal effective concentrations of AeCNMa in the low nanomolar range. Finally, we show that injections of AeCNMa into blood-fed mated female *Ae. aegypti* resulted in fewer eggs laid.

## 1. Introduction

Mosquitoes are significant vectors of human pathogens due to their requirement of blood meals to produce successive clutches of eggs. The process of egg production in mosquitoes involves the co-ordination of multiple events from pre-vitellogenic oogenesis [1], mating [2,3], host-seeking and blood feeding [4,5], blood digestion and yolk production [6,7], and oocyte maturation and oviposition [6,8,9,10]. At each stage, endocrine factors play critical roles in regulating these processes. In the pre-vitellogenic stage of development, juvenile hormone (JH) is secreted by the corpora allata shortly after eclosion [7,11]. JH ultimately acts upon the fat body and ovaries to induce competency for the uptake of yolk proteins (vitellogenin) synthesized from digested blood proteins [12]. Following a blood meal, insulin-like peptides (ILPs) and ovary ecdysteroidogenic hormone (OEH) are released from the mosquito brain, inducing ecdysteroid production in the ovaries and the expression of serine proteases in the midgut [4,6,9]. Ecdysteroids then act on the fat body to stimulate the expression of yolk protein genes, resulting in the production of vitellogenin, which is taken up by the 100–150 developing oocytes in the ovaries [4]. Disruption of any of these signaling pathways blocks the development of oocytes, resulting in reproductive failure [9,10].

Endocrine factors must transduce their signal across the membranes of target cells. Peptide hormones bind and activate transmembrane receptors, including G protein-coupled receptors (GPCRs), protein kinase receptors (PKRs), and receptor guanylyl cyclases (RGCs). The genome of the yellow fever mosquito, *Aedes aegypti*, encodes numerous GPCRs, PKRs, and RGCs, many of which have known functions and characterized ligands [13]. Ligands of some *Ae. aegypti* receptors are inferred through their orthology with characterized receptors in other arthropods, while others are true orphans with no known ligands [13]. Likewise, several neuropeptides identified in *Ae. aegypti* lack a characterized receptor [14].

The peptide CNMamide (CNMa) was initially discovered in *Drosophila melanogaster* and was named after the conserved carboxyl-terminal cysteine–asparagine–methionine motif that ends with an amide post-translationally modified from a glycine [15]. Two splice variants of the CNMa gene were predicted to be processed into two similar but distinct propeptides, which are subsequently cleaved into distinct peptides. The authors identified that the peptides were expressed in the brain and ventral nerve cord. The receptor for CNMa in *D. melanogaster* (DmCNMaR) was identified as a GPCR, CG33696, which is expressed in the *D. melanogaster* central nervous system (CNS). With a heterologous cell assay, it was demonstrated that DmCNMaR binds CNMa at nanomolar concentrations [15,16]. Initial studies showed no phenotype following transgenic RNAi knockdown of the receptor in *Drosophila*. Recently, Kim et al. [16] have reported the expression of *DmCNMa* in other tissues, including the midgut and fat body, and demonstrated that CNMa and CNMaR act as a signal of nutritional status and alter behavior in response to low essential amino acid levels in hemolymph.

Phylogenetic analysis of the CNMa gene indicated that it is widely conserved across arthropods, including in the crustacean *Daphnia pulex*, though it appears absent in Lepidoptera [15]. CNMaR has undergone multiple duplications, first into the paralogs CNMaR-1 and CNMaR-2, prior to the diversification of Hymenoptera. Paralogs of the receptor were differentially lost among insect orders, with all examined dipteran and lepidopteran genomes retaining the CNMaR-1 paralog but not CNMaR-2, though the red flour beetle, *Tribolium castaneum*, retained only the CNMaR-2 paralog. A second duplication event occurred in the Culicidae as two paralogs of CNMaR-1 are seen in the genomes of the African malaria mosquito, *Anopheles gambiae*, the common house mosquito, *Culex quinquefaciatus*, and *Ae. aegypti*, though the evolutionary timing of the duplication and its biological significance was not addressed [15]. The subsequent increase in available insect genomes and the improved annotation of existing genomes now allows for a more detailed assessment of the evolution of the receptor paralogs, including the extent to which different lineages retain multiple copies of CNMaR.

Using transcriptomic data, we determined that one *Ae. aegypti* paralog of DmCNMaR was highly expressed in reproductive tissues following a blood meal [17], suggesting a possible role for the peptide in the regulation of reproduction. We assessed the binding of AeCNMa to both paralogs of *Ae. aegypti* CNMaR-1 separately expressed in a mammalian cell line and found that the peptide activated both receptors but at slightly different concentrations. We next sought to identify a function for AeCNMa, by injecting it into blood-fed females and assessing whether reproductive processes were altered. Mated, blood-fed females injected with CNMa laid fewer eggs than control mosquitoes, indicating this peptide–receptor system may be involved in the regulation of oviposition by mosquitoes.

## 2. Materials and Methods

### 2.1. Mosquitoes

The UGAL strain of *Ae. aegypti* was used in all experiments. Mosquitoes were kept at 27 °C on a 16:8 h L:D cycle. Larvae were fed Cichlid goldfish pellets, and adults were provided with an 8% sucrose solution for 2 days post-eclosion. For virgin female experiments, individual pupae were placed in 0.5 mL of water in the wells of a 48-well polystyrene plate and returned to the environmental chamber to eclose. Following eclosion, adults were sorted by sex and kept in female-only containers prior to bioassays. Females were fed on defibrinated rabbit blood (Hemostat Laboratories, Dixon, CA, USA) provided via a water-jacketed parafilm membrane artificial feeder warmed to 37 °C.

### 2.2. Sequence Analyses

Orphan peptide hormone receptors identified by Vogel et al. [13] were queried against a developmental RNAseq dataset [17]. Receptors with highly differential expression in female reproductive tracts were selected for further study. *Ae. aegypti* orthologs of the DmCNMaR (CG33696) and DmCNMa peptide (CG13936) were identified through BLAST searches against the NCBI non-redundant database while limiting hits to arthropods (taxid: 6656). While the DmCNMa sequence has two splice forms, we only ever saw evidence of a single band when amplifying the complete AeCNMa sequence with PCR, and melting temperature analysis of AeCNMa never suggested the existence of alternative forms. Furthermore, no evidence of alternative splice forms was seen in the transcriptomic data supporting the gene model in VectorBase. To obtain sequences for phylogenetic analysis, AeCNMaR-1b (AAEL018316) was used as a query against the NR database, again restricting results to hits within Arthropoda. Additional sequences were identified through queries of OrthoDB [18]. Each sequence was assessed for the presence of the characteristic seven transmembrane domains of canonical GPCRs [19]. To identify potentially mis-annotated sequences, the genomic regions surrounding partial CNMaR sequences were aligned against the *Ae. aegypti* CNMaR-1a sequence with Exonerate [20] to identify the missing section of the sequence. The identified regions were then appended to the annotated sequences and the merged sequences were used for downstream phylogenetic analysis. Only sequences with six or more identified transmembrane domains were included in the analysis. CNMaR sequences were aligned using MAFFT with the -linsi option [21]. Alignments were trimmed using trimAl with a gap threshold of 0.4 [22]. The alignment was used as an input to RAxML [23] with the following options: -f a -x 1 -N 100 -p 1. Trees were visualized in Figtree (http://tree.bio.ed.ac.uk/software/figtree/, accessed on 25 November 2018).

### 2.3. Peptide Synthesis, Receptor Cloning, and GPCR Activation Assays

The CNMa peptide was synthesized and HPLC purified (>90% purity) by Royobiotechs (Shanghai, China) based on the published sequence of the AeCNMa peptide sequence [15]. Both the amidated (YMSLCHFKLCNMa) and the non-amidated (YMSLCHFKLCNM) were produced with a disulfide bridge between cysteines 5 and 10. The complete coding sequence of the two putative *Ae. aegypti* CNMa receptors (VectorBase genes AAEL024199 and AAEL018316, which we henceforth refer to as AeCNMaR-1a and AeCNMaR-1b, respectively) were commercially synthesized (Integrated DNA Technologies) and subsequently subcloned into a pcDNA3.1+ mammalian expression vector [24]. A Chinese hamster ovary cell line stably expressing the calcium-dependent bioluminescent protein aequorin (CHO-K1 aeq) [25,26] was grown in Dulbecco’s modified eagles medium: nutrient F12 (DMEM:F12) media containing 10% heat-inactivated fetal bovine serum (FBS; Wisent, St. Bruno, QC, Canada), 200 μg/mL geneticin, and an antimycotic-antibiotic mixture as described previously [27]. When cells reached 80–90% confluency, they were transfected using Lipofectamine 3000 transfection reagent following manufacturer recommendations (Life Technologies, Burlington, ON, Canada) to transiently express a mammalian construct in pcDNA3.1+ containing either of the two candidate CNMa receptors or mCherry (pcDNA3.1+ mCherry was a gift from Scott Gradia, Addgene plasmid #30125), the latter of which served as a negative control for the receptor functional assay. After 48 h following transfection, CHO-K1 aeq cells were harvested by dislodging from culture flasks using Dulbecco’s PBS containing EDTA and subsequently resuspended in receptor assay media [26].

Other *Ae. aegypti* peptides used in this study included “capability” (CAPA)-1 (GPTVGLFAFPRV-NH_2_), CAPA-2 (pQGLVPFPRV-NH_2_), AedaeCAPA-pyrokinin (PK) 1 (AGNSGANSGMWFGPRL-NH_2_), adipokinetic/corazonin-like peptide (ACP; pQVTFSRDWNA-NH_2_), adipokinetic hormone (AKH) (pQLTFTPSW-NH_2_), and corazonin (CRZ; pQTFQYSRGWTN-NH_2_) [24,26,27]. Serial dilutions of the peptide stocks were prepared in bovine serum albumin (BSA) assay media (DMEM:F12 media + 0.1% BSA + 1× antibiotic-antimycotic) to test concentrations ranging from 10 pM to 50 μM. Single concentrations of the various peptides were loaded into individual wells of white 96-well luminescence plates (Greiner Bio-One, Frickenhausen, Germany) with each peptide concentration tested in quadruplicate. CHO-K1 aeq cells expressing either of the two AeCNMa receptors or mCherry were prepared for the functional assay, as described above, and injected into each well of the 96-well luminescence plates by an automated injector unit integrated with the Synergy 2 Multi-Mode Microplate Reader (BioTek, Winooski, VT, USA). The kinetic luminescent response was measured for 10 s immediately following the application of the cells into the wells containing the different peptide treatments or the negative (i.e., BSA media alone) or positive (i.e., 50 μM ATP) controls as described previously [26]. Data was assembled in Microsoft Excel and then analyzed in GraphPad Prism 7.02 (GraphPad Software, San Diego, CA, USA) where the dose–response curves from multiple biological replicates were used to determine the half maximal effective concentrations (EC_50_).

### 2.4. QRT-PCR Analysis of Target Gene Expression

Cohorts of female *Ae. aegypti* were sampled 8 to 10 days post-eclosion, pre-blood meal (NBF), then at 2, 4, 6, 8, 12, 24, 48, and 72 h PBM. Four or more females from each timepoint were dissected in cold, RNase-free PBS into head, midgut, ovaries, and abdominal body wall and fat body (“pelt”). Tissue samples were immediately frozen and stored at −80 °C. Tissues were thawed on ice, homogenized with a rotor type pestle, and then total RNA was extracted using the RNeasy Mini kit (Qiagen, Venlo, The Netherlands) according to the manufacturer’s instructions. RNA was treated with DNase using the Turbo DNA-free kit (Ambion, Austin, TX, USA). cDNA was synthesized from 100 ng of RNA with the iScript cDNA synthesis kit (BioRad, Hercules, CA, USA) and used as template in qPCR using the Quantifast SYBR Green PCR kit (Qiagen). The primers for each product are listed in Table S1. qPCR was performed on the Qiagen Rotorgene as described in [28]. Absolute standard curves were produced by cloning each qPCR product into the pSCA vector using the Strataclone PCR cloning kit (Agilent, Santa Clara, CA, USA) and preparing plasmid standards of a known copy number. For comparisons between mating status, the copy number of each target was normalized to the copy number of ribosomal protein S7.

### 2.5. Bioassays

Three- to five-day-old mated females were blood-fed as detailed above, and groups were injected with a range of CNMa in 0.5 μL of saline or saline alone (AS) for a control. Females were allowed to recover and then housed individually in mesh cages. At 3 d PBM, a wet paper towel was provided to the females to facilitate egg laying for up to 48 h and then the number of eggs was counted. To assess possible interactions of CNMa with OEH and ILP3, mated females were blood-fed then decapitated and injected at 1 h PBM with 150 pmol of CNMa, OEH, or ILP3, or combinations of each hormone [29]. Ovaries were dissected at 24 h PBM and yolk deposition was measured along the anterior–posterior axis for five oocytes from each individual, using an ocular micrometer fitted to a dissecting microscope. For CNMa and JH experiments, pupae were isolated in eclosion chambers for 6 h and females were collected. Females were divided into two groups, one which was immediately injected with 150 pmol of CNMa or saline. The other half of the cohort was held for an additional 8 h then injected with the same solutions. At 3 d post-eclosion, females were blood-fed and oviposition was measured for all treatment groups. 

### 2.6. Proteomic Studies of Female and Male Tissues

To determine if CNMa was transferred from males to females, we examined different male and female tissues before and after mating for the presence of CNMa using HPLC and mass spectrometry. Fifty heads, thoraces, and abdomens from 3–5 day old *Ae. aegypti* females were collected separately in 200 µL of 80% acetonitrile/0.01% trifluoroacetic acid (TFA) and frozen at −80 °C overnight. Samples were thawed and sonicated at low power for 10 s with 100 µL 80% ACN/TFA added. Samples were centrifuged for 5 min at 21k RCF, and the supernatant was removed and lyophilized. Samples were resuspended in 500 µL of 10% ACN/TFA for 2 h then centrifuged as before. After a blank run, supernatant was injected into a Jupiter C18 column (300 Å, 4.5 × 200 mm) on a Beckman 126/166 chromatography unit. Samples were run in the following sequence: head extract, thorax extract, abdomen extract, 5 µg of AeCNM and AeCNMa. Sixty male accessory glands were collected from virgin *Aedes* males into 1 mL of 10% ACN/0.1% TFA and frozen at −80 °C. After thawing and sonification, the samples were centrifuged and injected onto the column and eluted as above. Sample material was eluted with a gradient (solvent A, pure water with 0.1%TF; solvent B, 80% ACN/0.1% TFA/pure water: 10%B to 80%B over 40 min; 1 mL/min), monitored at 206 nm, and collected in tubes as 1 mL fractions. CNMa and CNM eluted in fraction 25, so fractions 24 and 25 from the body part/tissue HPLC runs were pooled separately, lyophilized, and subjected to MALDI-TOF mass spectroscopy on a Bruker Autoflex (TOF) mass spectrometer (Billerica, MA, USA) at the University of Georgia Proteomics and Mass Spectrometry Facility, along with synthetic CNMa and CNM.

## 3. Results

### 3.1. CNMaR-1 Is Duplicated in the Culicidae

**Figure 1:**
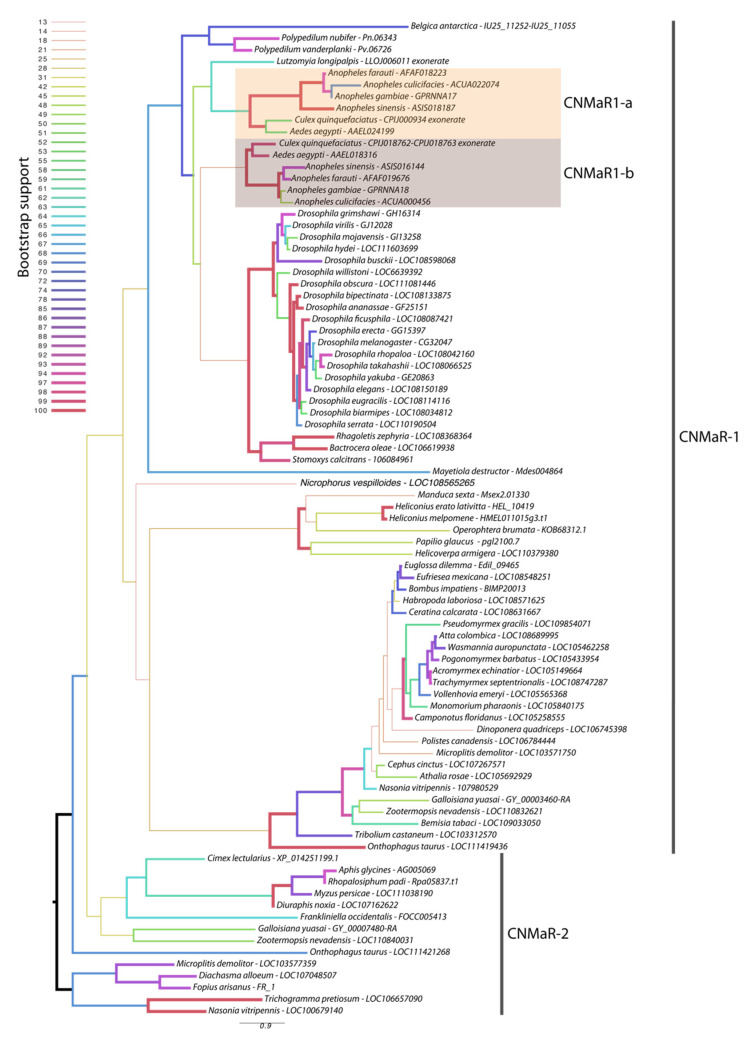
Maximum likelihood phylogenetic tree of *CNMaR* genes. At least two duplications of *CNMaR* have occurred in insects. The first duplication of *CNMaR* occurred prior to the divergence of the holo- and hemimetabolous insects as termites retain both CNMaR-1 and CNMaR-2 sequences. Sequences were aligned with MAFFT and phylogenetic tree constructed in RaXML with 100 bootstraps. Bootstrap support is listed at each node and is coded by branch color and weight as indicated by the legend. Sequence accession numbers are listed after each species name. Several sequences were obtained through alignment of coding sequences from closely related sequences with complete 7 transmembrane domains to genomic regions encoding partial sequences. These are indicated by tips labeled “exonerate”.

Our phylogeny leveraged additional insect genome sequences to further characterize the timing of CNMaR duplications and retentions or loss of orthologous sequences. Our analysis recovered two highly divergent CNMaR sequences in several insect lineages, including the Nevada dampwood termite *Zootermopsis nevadensis*, the milkweed bug *Onthophagus taurus*, and a large number of hymenopteran species (Figure 1). While there is not strong support for two monophyletic clades in our phylogeny, the distribution of CNMaR1 and CNMaR2 sequences suggests an ancient duplication followed by repeated, frequent losses. Our phylogeny also identifies two robustly supported clades of CNMaR-1 sequences in Culicidae. Due to limited genomic sequence availability, Jung et al. (2014) were unable to identify the members of each clade from *Ae. aegypti* and *C. quinquefaciatus*, but improvements in genome sequences and annotations for these species have clearly identified two copies of CNMaR-1 in these genomes, which we refer to as CNMaR-1a and CNMa-R1b. Our phylogenetic analysis lacked sufficient power and sampling to determine when the duplication occurred. Either CNMaR-1 duplicated prior to the divergence of the Brachycera, and CNMaR-1b was then lost in the Brachycera, or the duplication occurred after the split of Culicomorpha from Psychomorpha, but prior to the divergence of the Anopheline and Culicine lineages (Figure 1). The latter is the more parsimonious evolutionary trajectory, but the orthologs of CNMaR-1a group with sequences from Brachycera species, though this branch has very low support (18/100 bootstraps).

Orthologs of AeCNMaR-1a and AeCNMaR-1b were found in species across the family Culicidae. We did not include orthologs from every species with an available genome, as the sequences of CNMaR-1a/b were incomplete in many annotations. However, sequences containing all seven transmembrane domains were found in *Anopheles* spp. from across the phylogeny of sequenced genomes from this genus [30]. In some cases, we were able to identify exonic sequences that were potentially missed during annotation by comparing the full-length sequence of *AeCNMaR-1a/b* against the genomic regions encoding partial *CNMaR* sequences. We further interrogated available transcriptomic data for evidence of expression and found many of these sequences lack any evidence for expression. Therefore, it is possible that these are pseudogenes of recent origin.

The most closely related outgroups to Culicidae with available genomic sequence data are the phlebotomine sand flies *Lutzomyia longipalpis* and *Phlebotomus papatasi* (Diptera: Psychodomorpha: Psychodidae). BLAST and OrthoDB searches against the genomes of *P. papatasi* found no significant hits against the *Ae. aegypti* CNMaR-1b sequence, while the partial sequence of a single ortholog was found in *L. longipalpis* (Figure 1). All other dipteran genomes examined encoded a single ortholog of CNMa-1, suggesting that the duplication event occurred after the divergence of Psychomorpha from Culicomorpha.

### 3.2. CNMa Receptor Binding

**Figure 2:**
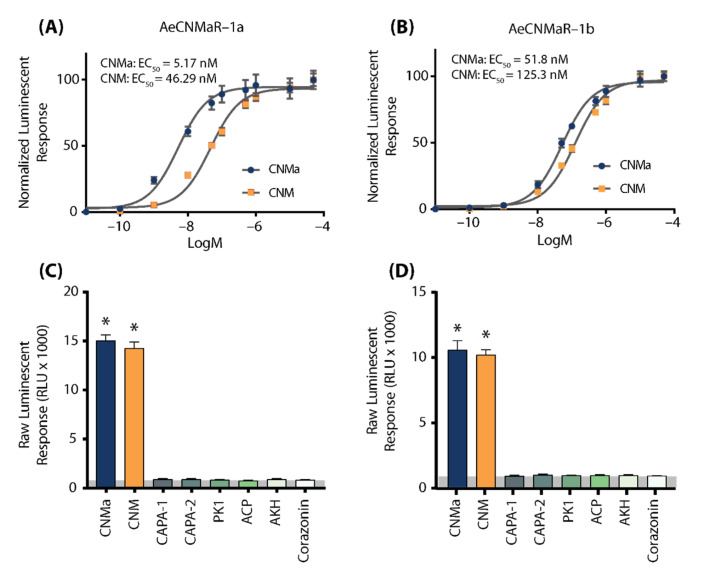
Luminescent response of CHO-K1 aeq cells expressing the *Ae. aegypti* CNMa receptors, AeCNMaR-1a (**A**,**C**) and AeCNMaR-1b (**B**,**D**). Transient expression of receptors in cells was used to examine functional activation by amidated and non-amidated CNM peptides. Both ligands were able to activate both receptors, with the AeCNMaR-1a receptor displaying higher sensitivity. Luminescence at every dose examined was normalized to the responses obtained following 50 µM AeCNMa application (**A**). Raw luminescent responses were also examined following treatment with various *Ae. aegypti* peptides tested at 1 µM (**C**,**D**). Only AeCNMa and AeCNM peptides were able to elicit a response significantly different from background control (BSA media alone in grey shaded area), denoted by an asterisk, as determined by a one-way ANOVA and Dunnett’s multiple comparison post-hoc test.

We next examined whether the two receptors bound CNMa in a similar manner. Heterologous functional analysis of the *Ae. aegypti* CNMaRs revealed a dose-dependent selective binding and downstream activation of luminescence signals by the AeCNMa ligand, with an EC_50_ value of 5.17 nM for AeCNMaR-1a (Figure 2A) and 51.8 nM for AeCNMaR-1b (Figure 2B). Both receptors were also responsive to the non-amidated AeCNM peptide, although the absence of C-terminal amidation resulted in lower potency, with EC_50_ values of 46.29 nM for AeCNMaR-1a (Figure 2A) and 125.3 nM for AeCNMaR-1b (Figure 2B). AeCNMaR-1a demonstrated a 9-fold increase in sensitivity for the amidated peptide (Figure 2A,C), whereas the AeCNMaR-1b receptor isoform displayed greater promiscuity for the two ligands, with only a 2.4-fold greater sensitivity for the amidated peptide (Figure 2B,D).

Neither of the receptors were responsive to several other *Ae. aegypti* peptides tested at 1 µM, which yielded luminescent responses similar to background levels when BSA media alone was applied (Figure S1). Instead, only AeCNMa and AeCNM were able to elicit a significant response several orders of magnitude over the BSA media background luminescent response (Figure S1).

To further verify that luminescence was specifically a result of AeCNM receptor binding and activation upon peptide application, cells were also transfected with pcDNA3.1^+^ encoding mCherry. No luminescence was detected in CHO K1 aeq mCherry-expressing cells in response to 1 µM AeCNMa or AeCNM (Figure S1) confirming that the AeCNMa- or AeCNM-induced luminescent response required the expression of either of the two AeCNMa receptors.

### 3.3. CNMa Peptide and Receptor Expression

**Figure 3:**
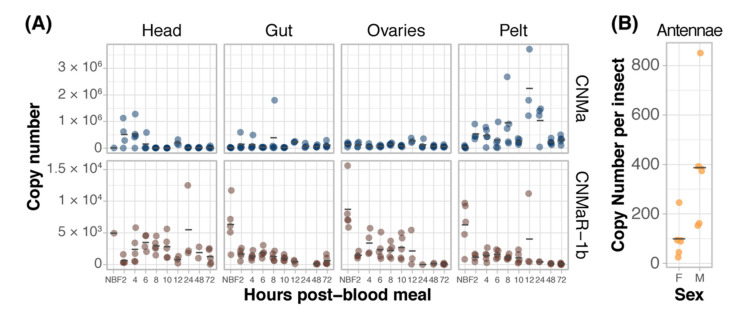
Expression patterns of AeCNMa receptors and peptide. (**A**) qPCR was used to measure *AeCNMaR-1a* (yellow), *AeCNMaR-1b* (red), and *AeCNMa* (blue) expression in tissues dissected from adult females at various times post-blood meal. Expression of *AeCNMaR-1b* was highest in ovaries prior to a blood meal (NBF), decreasing steadily until 12 h PBM. Expression of the receptor was also high in other tissues prior to a blood meal, and there was moderate expression in female heads 4–10 h PBM. *AeCNMa* was most highly expressed in female pelts 2–24 h post blood meal, peaking at 12 h PBM, with secondary expression in the head at 2–4 h PBM. (**B**) *AeCNMaR-1a* expression was only detected in male antennae. For all plots, points represent individual expression values and bars represent mean.

We examined the expression of *AeCNMa*, *AeCNMaR-1a*, and *AeCNMaR-1b* across life stages and in female tissues taken at various times post-blood meal (PBM). Expression of *AeCNMaR-1a* was never detected in any juvenile-stage or female samples. *AeCNMa* and *AeCNMaR-1b* were expressed at varying levels across tissues and development. We found elevated *AeCNMa* expression at 2–4 h PBM in heads (F_9,32_ = 3.36, *p* = 0.0052, ANOVA) and in pelts beginning at 2 h PBM, peaking at 12 h PBM, and returning to low levels by 48 h PBM (Figure 3A, F_9,36_ = 5.19, *p* = 0.0002, ANOVA). The *AeCNMaR-1b* copy number was highest in the tissues of non-blood-fed (NBF) females (F_38,119_ = 4.28, *p* < 0.0001, ANOVA, *p* < 0.05, Tukey’s HSD), which typically decreased within a few hours after blood feeding, and later was more variable or increased in heads, ovaries, and pelts, but not guts. We also examined the expression of *AeCNMa* and *AeCMaR-1b* in larvae and pupae, finding that both transcripts were at low levels in all juvenile life stages relative to adult expression (Figure S2).

**Figure 4:**
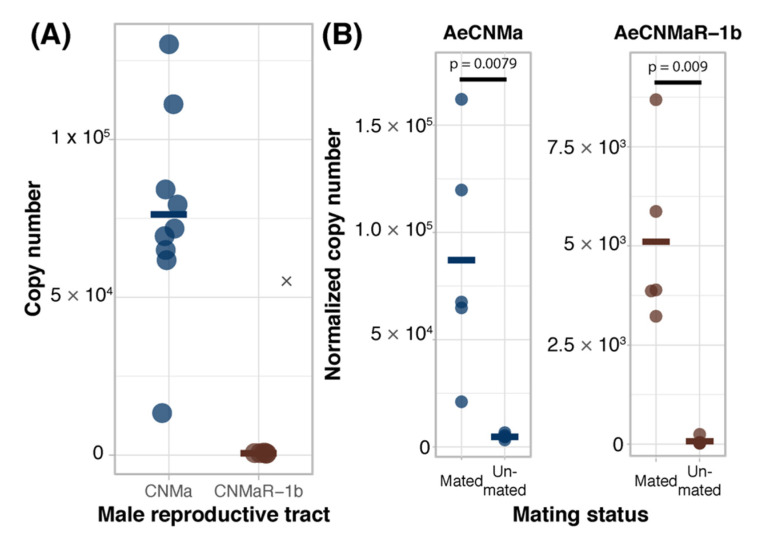
Ligand and receptor expression in male reproductive tissues and induction of *AeCNMa* and *AeCNMaR-1b* in females by mating. (**A**) *AeCNMa* was expressed at high levels in male reproductive tracts (testes and accessory glands), while expression of the receptor *AeCNMaR-1b* was negligible. (**B**) Mating of virgin females induced expression of *AeCNMa* (*p* = 0.0079, Wilcoxon signed-rank test) and *AeCNMaR-1b* (*p* = 0.009, Wilcoxon signed-rank test).

We expanded our expression analysis to include males, as previous transcriptomic datasets suggested that *AeCNMaR-1a* was expressed in male antennae [31,32]. We confirmed that *AeCNMaR-1a* is expressed in male antennae and was not detected in other tissues examined (Figure 3B). We found that *AeCNMa* was highly and specifically expressed in male accessory glands, but *AeCNMaR-1b* was not (Figure 4A). We next asked whether the expression of *AeCNMaR-1b* or *AeCNMa* was influenced by the mating status of females. We found that mated females had a significantly higher abundance of *AeCNMa* and *AeCNMaR-1b* than un-mated females (Figure 4B, *p* = 0.0079 and *p* = 0.009, Wilcoxon rank–sum test). We attempted to silence the expression of *AeCNMaR-1a* through injection of dsRNA into the thorax of female mosquitoes. While this approach successfully silenced the expression of other peptide hormone receptors in *Ae. aegypti* [10], we were unable to reduce expression of the *AeCNMaR-1b* or *CNMa* transcript through traditional RNAi (data not shown).

### 3.4. Effects of AeCNMa Injection on Ae. aegypti Reproduction

**Figure 5:**
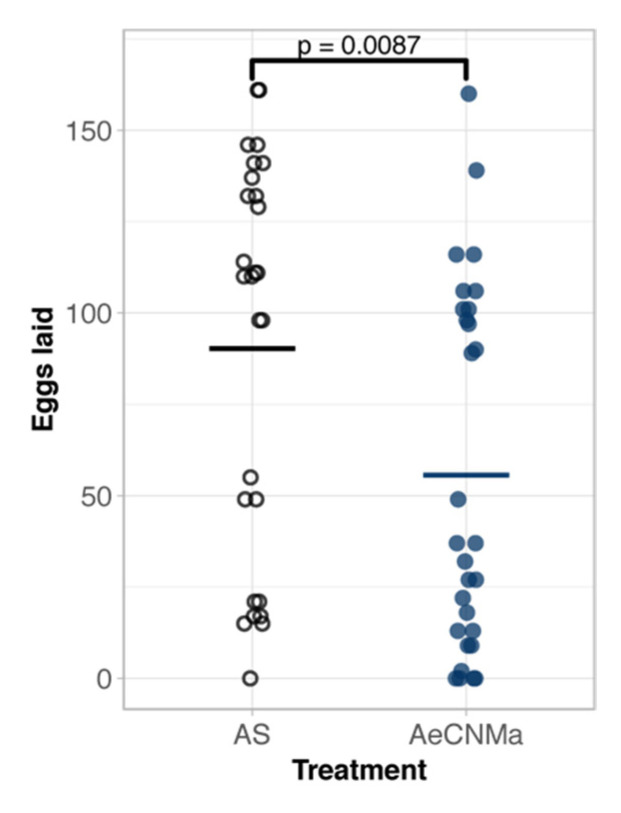
AeCNMa reduces egg clutch size in mated *Ae. aegypti* females. Age-matched females were blood fed on an artificial membrane feeding apparatus and immediately injected with CNMa or Aedes saline (AS). After three days females were allowed to oviposit and eggs were counted 48 h later. Points represent individuals and bar represents mean. Females injected with AeCNMa produced significantly fewer eggs than controls (*p* = 0.0087, Wilcoxon rank-sum test).

Our expression analysis revealed a peak of *AeCNMaR*-1b in ovarian tissue in non-blood-fed females, followed by a steep decrease in expression following a blood meal. We therefore reasoned that CNMa may regulate some aspect of blood meal digestion or reproduction. To investigate this, we injected mated, blood-fed females with 150 pmol of AeCNMa (the optimal dose based on dose–response assay) immediately PBM (Figure 5). Mated females injected with AeCNMa laid significantly fewer eggs than controls. 

### 3.5. Presence of CNMa in Male Accessory Glands

The reduced oviposition of mated blood-fed females injected with AeCNMa, along with the high level of *AeCNMa* transcript abundance in the male reproductive tract and in mated females, suggested that AeCNMa may be a negative signal for oviposition during early vitellogenesis. We extracted male accessory glands for HPLC separation. Fractions eluting at the same time as the AeCNMa and AeCNM standards were subjected to mass spectroscopy. Neither peptide was detected in fractions from the extracts, suggesting that males do not transfer AeCNMa to females during copulation.

### 3.6. Interaction of CNMa and Other Reproductive Hormones

**Figure 6:**
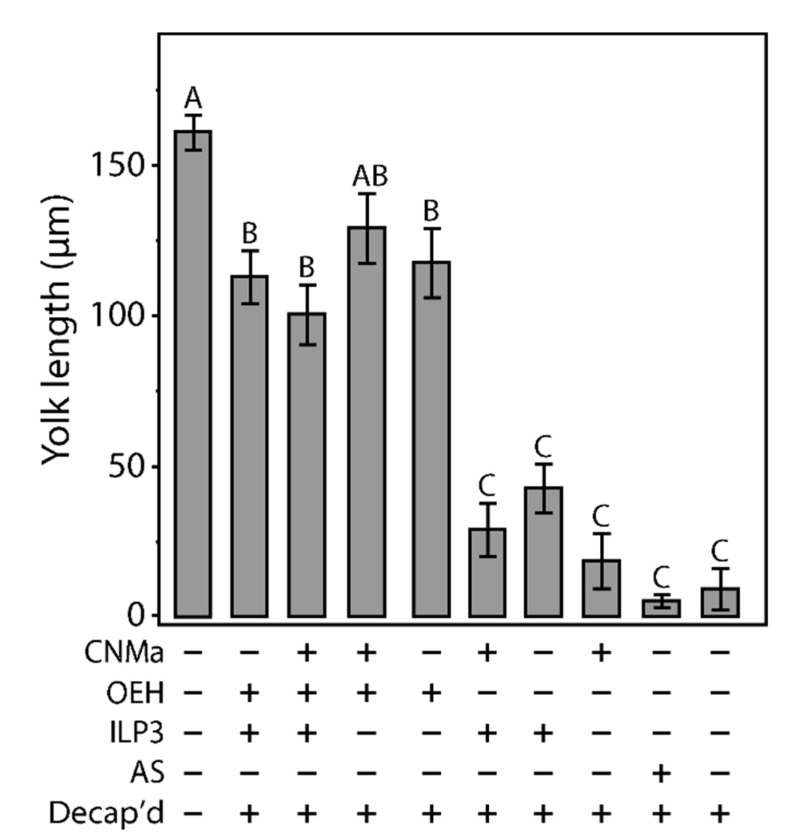
Injection of CNMa along with ILP3 or OEH does not have a significant effect on yolk uptake in decapitated females. Average amount of yolk per oocyte in mated, blood fed, decapitated females injected with combinations of CNMa (150 pmol), ILP3 (20 pmol), and OEH (20 pmol), Aedes saline (AS), or not injected (intact or decapitation only) at one-hour post blood meal. Error bars show one standard error above and below the mean. Yolk uptake in the CNMa only treatment was not significantly different from decapitated only and AS control treatments. There was no significant difference in yolk uptake in CNMa + OEH + ILP3 injected females compared to ILP3 + OEH injected females. Decapitated females injected with OEH + CNMa demonstrated no significant difference in yolk uptake relative to OEH only or OEH + ILP3 treatments. There was no significant difference in yolk uptake among ILP3 and OEH, ILP3 only, and CNMa and ILP3 treatments. Bars not connected by the same letter are statistically significantly different, *p* < 0.05, Tukey’s HSD.

Given that AeCNMa suppressed egg production by mated female mosquitoes, we next investigated whether the influence of AeCNMa on *Ae. aegypti* egg production was related to interactions with AeOEH or AeILP3 [10,33,34]. Decapitation shortly after a blood meal blocked endogenous OEH and ILP3 release from the brain, and injection of AeOEH or AeILP3 restored egg development. We found that AeCNMa did not affect yolk deposition elicited by OEH, ILP3, or both peptides together (Figure 6). These results, along with the early window of CNMa action in mated females, suggested that the role of the peptide was independent of OEH and ILP3.

**Figure 7:**
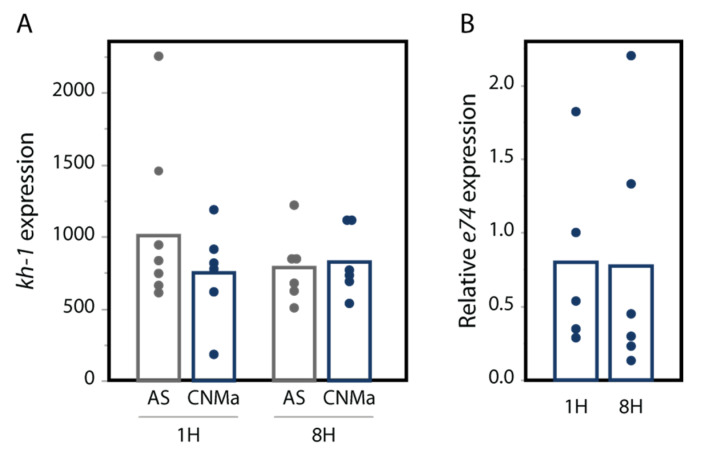
CNMa does not influence expression of (**A**) the JH-responsive gene *kh-1* or (**B**) the 20-hydroxyecdysone gene *e74*. qPCR was performed for kh-1 as described in the methods. For *e74*, expression is relative to *e74* expression in AS-treated insects. Treatment of insects with CNMa did not significantly impact expression regardless of time administered or gene tested (MANOVA *e74*: F_3,19_ = 0.8192, *p* = 0.50; *kh-1*: F1,20 = 0.5141, *p* = 0.68).

We also tested whether an injection of AeCNMa peptide into females resulted in changes in transcript abundance in the JH-responsive gene *kruppel-homolog 1 (kh-1)* or the ecdysteroid-responsive gene *e74*. We found no significant difference in their expression between female mosquitoes injected with AeCNMa or saline (Figure 7).

## 4. Discussion

The receptor of CNMa has repeatedly duplicated in insects, being differentially lost among lineages. Dipterans retain CNMaR-1, which duplicated prior to the divergence of Culicomorpha. The retention of both copies of the CNMaR-1 receptors in most mosquito lineages suggests that both genes encode functional proteins that are either redundant or have diverged in their functions. Expression patterns of the two receptors in *Ae. aegypti* differ markedly. *AeCNMaR-1a* expression was only detected in male antennae, with transcripts never detected in other tissues or timepoints. In contrast, *AeCNMaR-1b* expression was found primarily in ovary tissues prior to a blood meal, though lower levels of expression were detected in other tissues and life stages.

The gene encoding the peptide does not seem to have been duplicated, with only a single gene encoding a CNMa peptide in *Ae. aegypti* (AAEL010529) and the available *Anopheles* genomes [30]. The *Ae. albopictus* Foshan genome sequence [35] does encode two genes which yield identical CNMa peptides (AALF020855, AALF023040), though given the near identity of the mRNA sequences, their location on different scaffolds, and the incomplete nature of the *Ae. albopictus* genome sequence, it is possible that the two sequences are an artifact of genome mis-assembly. *AeCNMa* expression in *Ae. aegypti* females was detected primarily in abdomen pelts, which include the ventral ganglia, and, to a lesser degree, in heads with brains. Neurons or neurosecretory cells in these ganglia are likely sources of CNMa as in *D. melanogaster*, where DmCNMa was localized to tens of cells in the CNS [15] and in the gut in response to amino acid starvation [16].

Both AeCNMa receptors exhibited specific activation by the AeCNMa peptide, further supporting the hypothesis that both genes encode functional receptors. Surprisingly, AeCNMaR-1a had a higher affinity for AeCNMa than AeCNMaR-1b by approximately an order of magnitude, despite the broader transcript distribution and higher abundance of *AeCNMaR-1b*. Although AeCNMaR-1a had a higher affinity for AeCNMa, the EC_50_ of AeCNMaR-1b was still within the physiological range (~50 nM). While AeCNMa induced a stronger activation of both receptors than AeCNM, the non-amidated peptide still stimulated both receptors, though the EC_50_ was 1/10th and 1/5th lower than the amidated form for AeCNMaR-1a and 1b, respectively. Other studies illustrated that activation of the *D. melanogaster* and *Apis mellifera* CNMaRs with the *D. melanogaster* CNMa peptide was comparable to AeCNMaR-1a, while the *Bombyx mori* receptor EC_50_ was far higher (>1000 nM) than that of either *Aedes* receptor. Differences in AeCNMa binding affinity and in the expression patterns of the two receptors suggest that the peptide and receptors may have distinct functions in male and female mosquitoes. Future studies will examine the role of this peptidergic system in male-specific behaviors.

Given the tissue-specific distribution and timing of *AeCNMaR-1b* and *AeCNMa* expression, we suspected that AeCNMa and AeCNMaR-1b played a role in mosquito reproduction. We found that mating induced expression of *AeCNMa* in female mosquitoes, and thus we focused our experiments on determining the role of the peptide in reproduction in female mosquitoes. By injecting AeCNMa into female mosquitoes, we found that the peptide reduced the number of eggs laid by mated, blood-fed females. This effect was independent of the well-characterized effects of other hormones, specifically OEH, ILP3, and 20-hydroxyecdysone [10,34,36,37]. The effects of AeCNMa injection into female mosquitoes suggest that this peptide may play a role in signaling mating status. We examined whether AeCNMa was transferred from males to females during mating but did not detect the peptide in male accessory gland extracts.

CNMa has been shown to affect nutrient sensing in adult *D. melanogaster*. Low levels of amino acids in the gut stimulate DmCNMa expression via targeting rapamycin kinase (TOR) signaling. Specific microbes in the gut appear to regulate free amino acid levels, which then stimulate TOR signaling and ultimately CNMa production. The *D. melanogaster* CNMa receptor is predominantly found in the CNS, where the binding of CNMa drives a preference for diets rich in essential amino acids [16]. TOR signaling is also critical in the co-ordination of blood meal digestion and reproduction in *Ae. aegypti* [38], and future studies will examine whether nutrient signaling through TOR are important for the action of CNMa.

## Data Availability

Publicly available datasets were analyzed in this study. This data can be found here: www.vectorbase.org (accessed on 5 January 2022).

## Supplementary Materials

The following supporting information can be downloaded at: https://www.mdpi.com/article/10.3390/insects13030230/s1: Table S1: Sequences of primers used in the study; Figure S1: Validation of receptor activation by CNMa; Figure S2: *AeCNMa* and *AeCNMaR-1b* expression in larvae and pupae; Figure S3: Illustration of yolk-length measurement.
