# Supplementary material for: The Peptide Hormone CNMa Influences Egg Production in the Mosquito Aedes aegypti
Source: Insects. 2022 Feb 25;13(3):230. doi: 10.3390/insects13030230 (PMC8955854; doi:10.3390/insects13030230)
Supplement: Supplementary file 1 [file insects-13-00230-s001.zip › table S1.pdf]

Table S1: Primers used in this study

Target gene	Vectorbase accession	Purpose	Forward primer	Reverse primer
AeCNMa	AAEL010529	qPCR	CTTTTGCAAATTGTGGGAAAC	AAAGTTTTGACAGGGTTCTCT
		RNAi	TAATACGACTCACTATAGGGAGATGATTACACATTGGAGCTGC	TAATACGACTCACTATAGGGAGAAGTTCCAGAAGCTCAAGTAC
AeCNMaR-1a	AAEL024199	qPCR	TATTACACTCCAGCACTGGTC	GCCAGGTAATAAGATGAGGAT
		RNAi	TAATACGACTCACTATAGGGAGAAGTTGGACTTACCTTTCTTGG	TAATACGACTCACTATAGGGAGACTTCCCGTACATCACAGATAG
AeCNMaR-1b	AAEL018316	qPCR	GCCATGAATGAAACCATCTG	GTAAATGGAACCACGAACAC
		RNAi	TAATACGACTCACTATAGGGAGAGTAGTTACGACATCGATCAGC	TAATACGACTCACTATAGGGAGAGCTGATAAACACTCCAATCAG

Underlined sequences denote gene-specific portion of dsRNA synthesis primers
