# Supplementary figures and images for: The Peptide Hormone CNMa Influences Egg Production in the Mosquito Aedes aegypti
Source: Insects. 2022 Feb 25;13(3):230. doi: 10.3390/insects13030230 (PMC8955854; doi:10.3390/insects13030230)

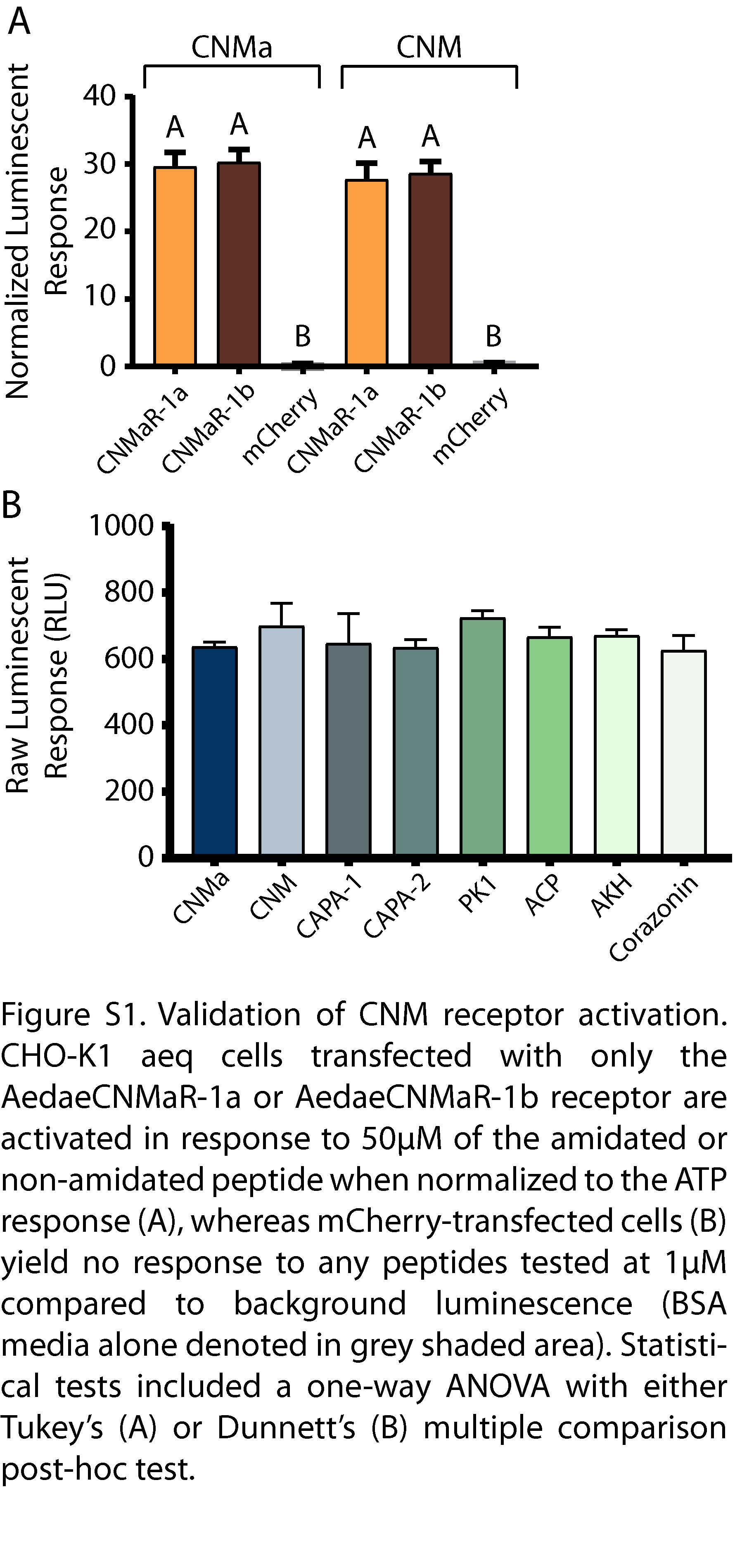

Supplement: Supplementary file 1 [file insects-13-00230-s001.zip › Figure S1 - Addtional binding.png]

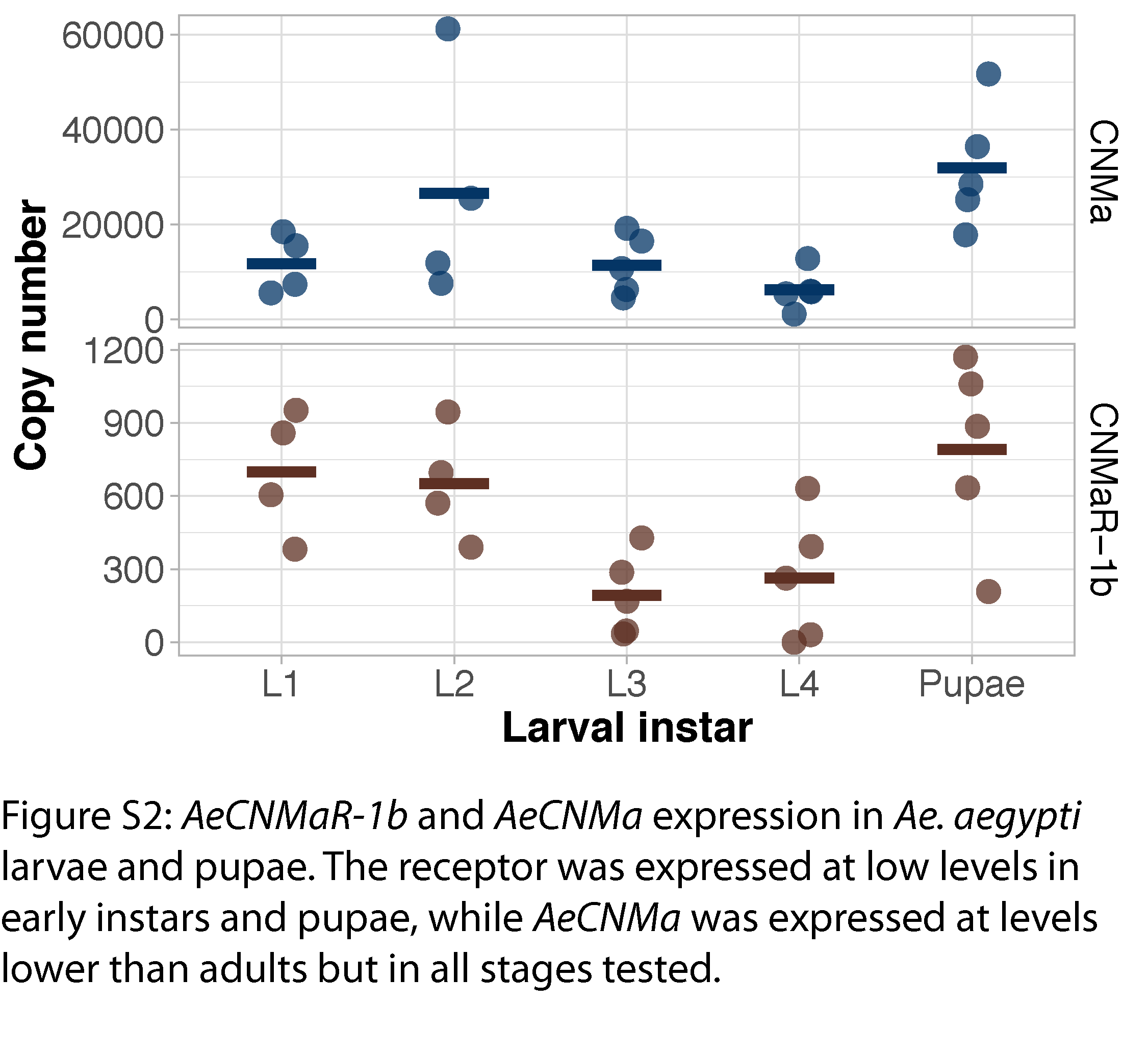

Supplement: Supplementary file 1 [file insects-13-00230-s001.zip › Figure S2 - larval expression.png]

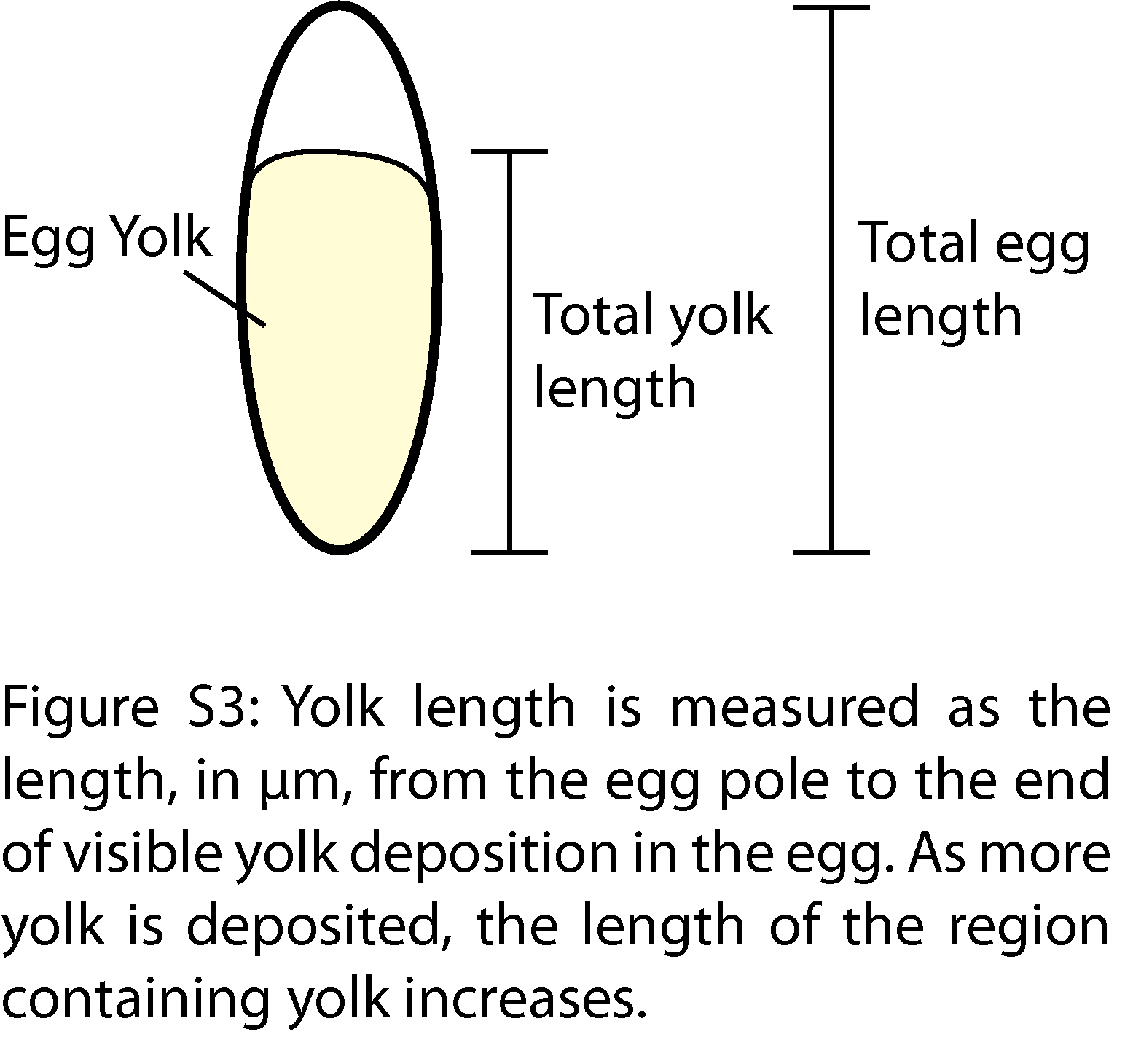

Supplement: Supplementary file 1 [file insects-13-00230-s001.zip › Figure S3 - Yolk length.png]
